# Microcomputed tomography: approaches and applications in bioengineering

**DOI:** 10.1186/scrt534

**Published:** 2014-12-29

**Authors:** Joel D Boerckel, Devon E Mason, Anna M McDermott, Eben Alsberg

**Affiliations:** Department of Aerospace and Mechanical Engineering, Tissue Engineering and Mechanobiology Laboratory, Bioengineering Graduate Program, University of Notre Dame, 142 Multidisciplinary Research Building, Notre Dame, IN 46556 USA; Department of Biomedical Engineering, Wickenden Bldg, Room 204, 10900 Euclid Ave, Cleveland, OH 44106 USA

## Abstract

Microcomputed tomography (microCT) has become a standard and essential tool for quantifying structure-function relationships, disease progression, and regeneration in preclinical models and has facilitated numerous scientific and bioengineering advancements over the past 30 years. In this article, we recount the early events that led to the initial development of microCT and review microCT approaches for quantitative evaluation of bone, cartilage, and cardiovascular structures, with applications in fundamental structure-function analysis, disease, tissue engineering, and numerical modeling. Finally, we address several next-generation approaches under active investigation to improve spatial resolution, acquisition time, tissue contrast, radiation dose, and functional and molecular information.

## Introduction

**Figure 1:**
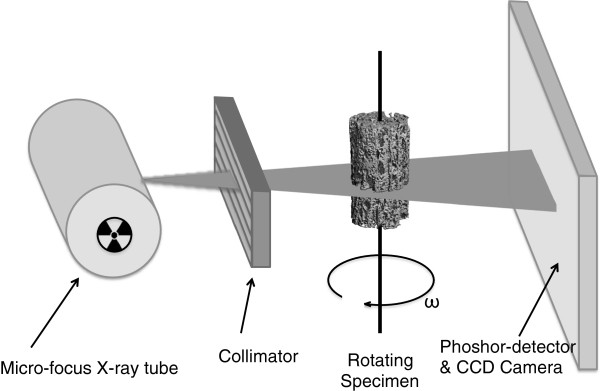
**Principal components of a microcomputed tomography scanner.** A micro-focus x-ray tube, or synchrotron emitter for monochromatic beam generation, produces radiation, which is collimated and passed through the object. The radiation is attenuated by the sample, and this attenuation is measured by a charge-coupled device (CCD) camera with a phospholayer coating to convert x-rays to visible light. A three-dimensional rendering of the sample is achieved by scanning at different angles of rotation and reconstructing through transformation of two-dimensional projections.

Microcomputed tomography (microCT or μCT) is a non-destructive imaging tool for the production of high-resolution three-dimensional (3D) images composed of two-dimensional (2D) trans-axial projections, or ‘slices’, of a target specimen. MicroCT equipment is composed of several major components: x-ray tube, radiation filter and collimator (which focuses the beam geometry to either a fan- or cone-beam projection), specimen stand, and phosphor-detector/charge-coupled device camera (Figure 1). Reconstruction of a 3D image is performed by rotating either the sample (for desktop systems) or the emitter and detector (for live animal imaging) to generate a series of 2D projections that will be transformed to a 3D representation by using a digital process called back-projection [1, 2]. This non-destructive imaging modality can produce 3D images and 2D maps with voxels approaching 1 μm, giving it superior resolution to other techniques such as ultrasound and magnetic resonance imaging (MRI) [2].

The principle of microCT is based on the attenuation of x-rays passing through the object or sample being imaged. As an x-ray passes through tissue, the intensity of the incident x-ray beam is diminished according to the equation, I_x_ = I_0_e^−μx^, where I_0_ is the intensity of the incident beam, x is the distance from the source, I_x_ is the intensity of the beam at distance x from the source, and μ is the linear attenuation coefficient [3]. The attenuation therefore depends on both the sample material and source energy and can be used to quantify the density of the tissues being imaged when the reduced intensity beams are collected by a detector array.

## Historical context

In 1979, Allan Cormack and Godfrey Hounsfield were awarded the Nobel Prize in Physiology or Medicine for the development of computer-assisted tomography and, by the late 1970s, clinical computed tomography (CT) was in widespread use; however, these systems were limited in resolution and yielded only 2D reconstructions as they relied on line x-rays and linear array detectors. In the early 1980s, Ford Motor Company physicist Lee Feldkamp developed the first microCT system to evaluate structural defects of ceramic automotive materials. Expanding on the concepts of clinical CT systems, Feldkamp conceived of using a cone-beam x-ray source and 2D detector and rotating the sample itself through 360°. He then developed the cone-beam algorithm to reconstruct fully 3D images from those projections [4]. A serendipitous meeting between Feldkamp and Michael Kleerekoper of Henry Ford Hospital led to the first scan of bone tissue, an iliac crest biopsy, and resulted in the first public evidence of microCT: an abstract from the 1983 meeting of the American Society for Bone and Mineral Research [5].

That same year, through connections at Henry Ford Hospital, Feldkamp was introduced to Steven Goldstein, an orthopedic biomechanician at the University of Michigan. Goldstein would name the technique ‘microcomputed tomography’, and this collaboration led to the first publication of microCT analysis of bone architecture, an evaluation of subchondral bone in experimental osteoarthritis [6]. This was followed shortly by the now well-known initial trabecular bone microstructure article [7]. In 1984, Goldstein replicated the Feldkamp microCT system in his laboratory, establishing the first university microCT system, and in the ensuing years it became clear that microCT would revolutionize the fields of bone biology and biomechanics. Several commercial microCT systems are now available worldwide, and new innovations continue to expand its speed, resolution, and applicability to non-mineralized tissues.

This review discusses microCT approaches for quantitative evaluation of bone, cartilage, and cardiovascular structures, with applications in fundamental structure-function analysis, disease, tissue engineering, and numerical modeling, and addresses next-generation systems under active investigation and development.

## Bone structure, disease, and adaptation

**Figure 2:**
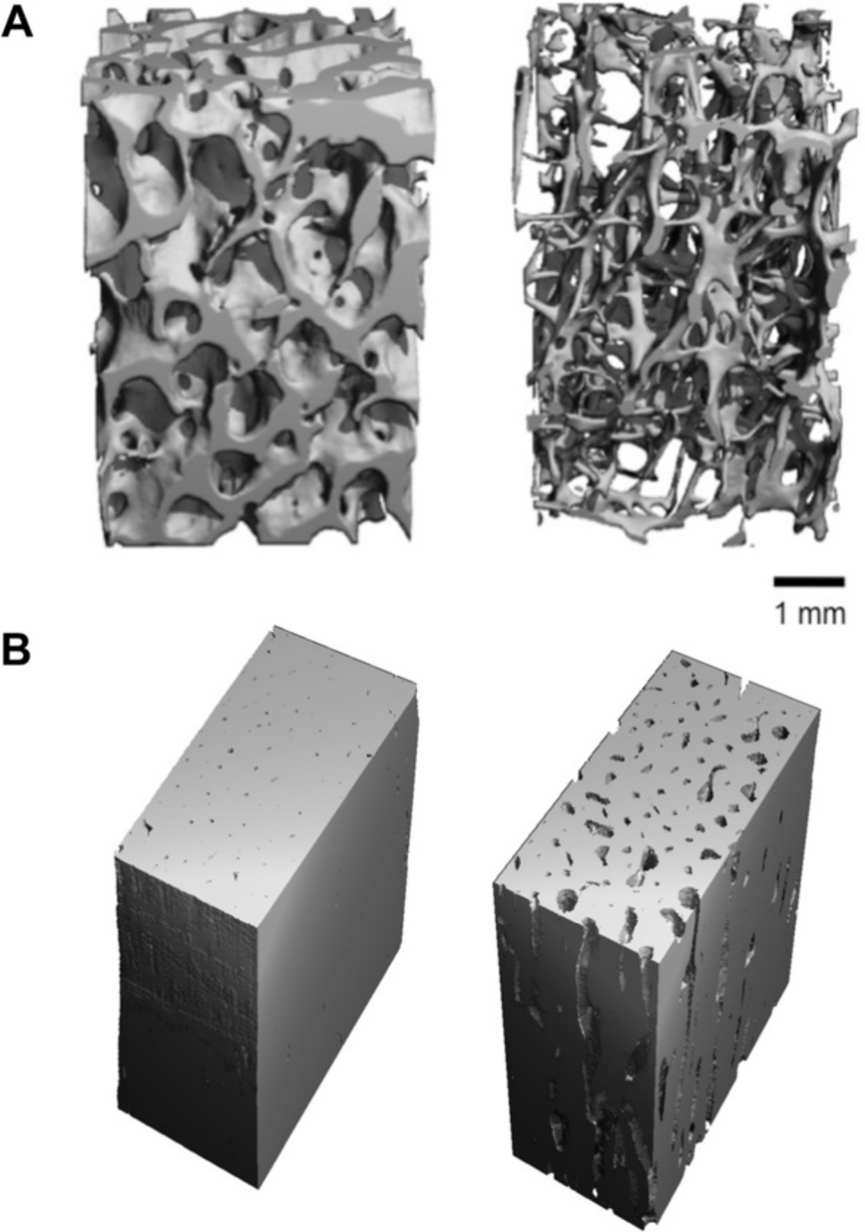
**Microcomputed tomography (microCT) reconstruction of cortical and trabecular bone.** MicroCT enables high-resolution three-dimensional reconstruction of microstructural characteristics from trabecular architecture to cortical porosity. **(A)** Trabecular bone from femoral neck of 51-year-old male (left) and 84-year-old female (right). **(B)** Diaphyseal femoral cortical bone of 18-year-old male (left) and 73-year-old female (right). Age, gender, disease, and other factors influence the microstructural properties of both cortical and trabecular bone, and these can be evaluated quantitatively by microCT.

**Figure 3:**
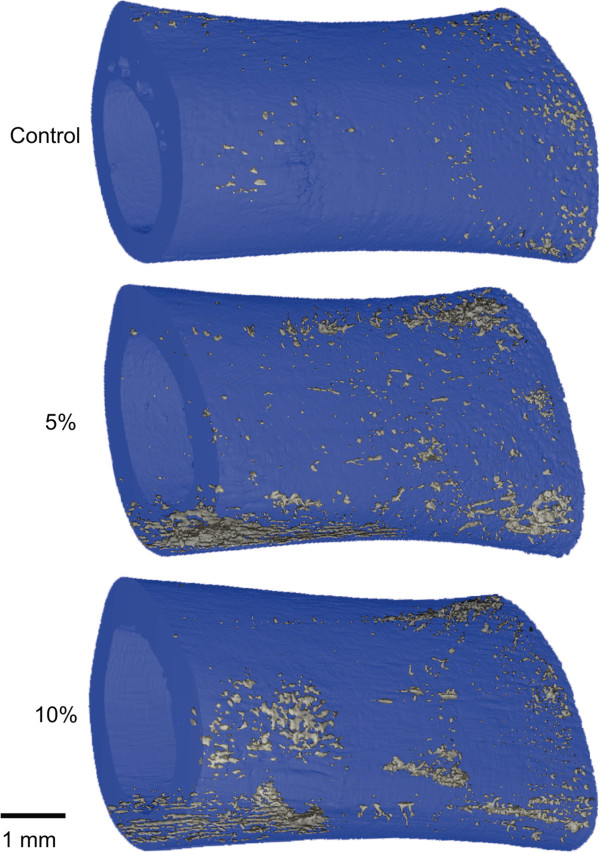
**Contrast-enhanced imaging of mechanical load-induced bone microdamage in rat femora.** Samples were loaded in three-point bending to 5% or 10% reduction in secant modulus and stained by barium sulfate (BaSO_4_) precipitation. Load-induced microcracks provide nucleation sites for barium and sulfate ions to accumulate. BaSO_4_ signal featured a linear attenuation coefficient approximately three times that of rat cortical bone, enabling co-registration of microdamage by microcomputed tomography. Reproduced with permission from Elsevier [10].

MicroCT is established as an essential tool for evaluating bone structure and quality and has been used to study metabolic bone diseases such as osteoporosis (Figure 2), to evaluate preclinical models of disease [8], and to test the efficacy of anti-resorptive and anabolic therapeutics, such as bisphosphonates [9]. One emerging technique for microCT-based evaluation of bone fragility induced by loading, aging, or osteoporotic disease is the use of contrast agents to detect and quantify bone microdamage. For example, barium sulfate (BaSO_4_) allows 3D assessment of exposed calcium to quantify fatigue microdamage in bovine trabecular (Figure 3) [10, 11].

MicroCT is now also a standard technique for evaluation of genomic factors on bone phenotype through the use of genomic and tissue-specific knockout mice, as reviewed elsewhere [12, 13]. For example, Wang and colleagues [14] used microCT analysis of both bone and vascular structures to show that deletion of von Hippel-Lindau, which regulates expression of the angiogenic growth factor vascular endothelial growth factor through modulating hypoxia-inducible factor (HIF)1α degradation, resulted in exceedingly dense, highly vascularized long bones, but normal calvariae, whereas the HIF1α knockouts had the opposite long-bone phenotype. Interestingly, the double knockout exhibited increased long-bone formation and vascularization, enabling identification of a compensatory function of the HIF2α subunit.

MicroCT imaging affords unique capabilities for non-destructive reconstruction of microstructural features, enabling approaches such as finite element (FE) analysis to evaluate local biomechanical behavior under complex loading conditions. This method allows virtual recapitulation of experimental or physiologic boundary conditions to estimate local stresses and strains within a tissue of complex geometry [15]. Important considerations for accurate FE analysis of biological tissues include mesh formulation and resolution, constitutive models that recapitulate salient features of tissue behavior, appropriate boundary conditions, and model size and convergence. Mesh generation may be accomplished either through custom, specimen-specific meshes featuring smooth boundaries and unstructured grids or through direct conversion of digital voxels to hexahedral brick elements. Direct, digital FE models are the easiest to create but may be limited by large model sizes and inaccuracies or instabilities at model or material boundaries, requiring at least four digital FEs through a beam cross-section for accuracy [16, 17].

Appropriate constitutive model selection is critical for analysis of biological materials, including bone. Although numerous constitutive formulations have been employed, a universally applicable model has not been identified, and formulation should be carefully considered for each application. For example, some approaches account for inhomogeneity by scaling the local Young’s modulus or ultimate stress with microCT-measured local density, either linearly or, more accurately, using a power-law relationship [18–20]. Others have coupled non-linear local constitutive models with microCT-based FE models to predict local plasticity and macroscopic failure of trabecular bone and to relate bone microarchitectural features with apparent-level mechanical behavior [21, 22]. Intrinsic mechanical properties can also be validated directly through local measurement by nanoindentation [22] or at the effective level by comparison and scaling with mechanical tests [23]. Finally, accurate physiological boundary conditions are frequently difficult to quantify but may be estimated by inverse dynamics, direct muscle force measurement by electromyography, scaling muscle reaction forces with muscle length or physiological cross-sectional area, or through objective optimization approaches [24].

**Figure 4:**
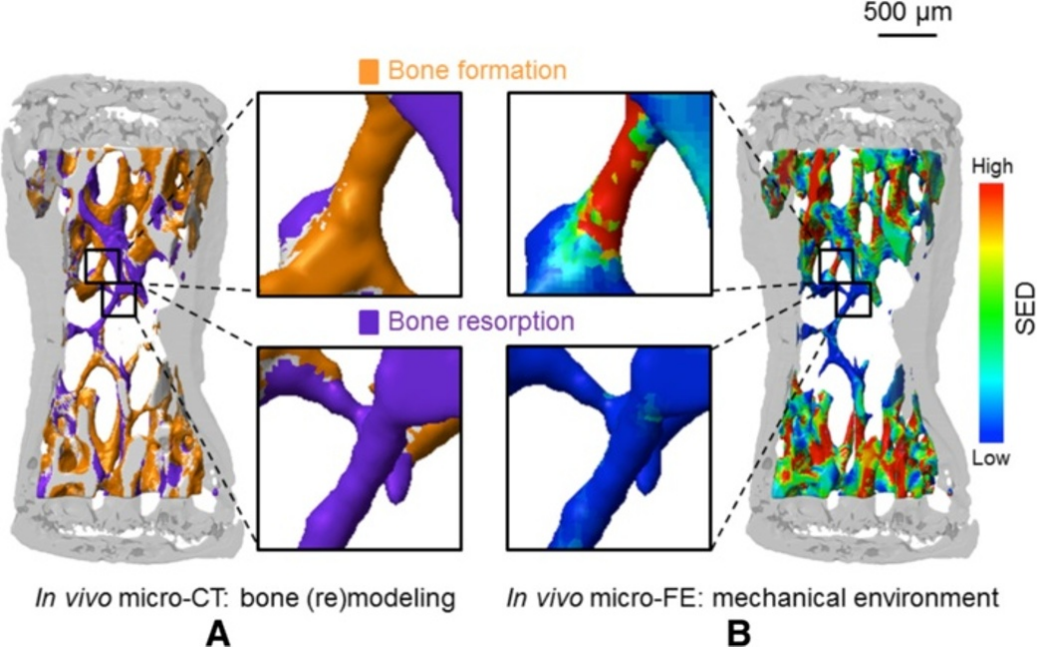
**Correlation of local tissue strains with regions of bone formation and resorption by longitudinal** ***in vivo*** **microcomputed tomography (microCT) and finite element (FE) analysis.** The sixth caudal vertebra of mice were loaded by pinned compression of the fifth and seventh vertebrae, resulting in a cyclic load of 9 N. Serial, co-registered microCT scans were analyzed to determine locations of bone formation and resorption **(A)** and correlated with locations of high/low strain energy density (SED) **(B)**, calculated by FE analysis. Bone formation and resorption were significantly more likely in regions of high and low SED, respectively. Reproduced with permission from PLOS [39].

Current applications of microCT-based FE modeling include evaluation of bone quality, microdamage and failure [25–27], effects of mechanical stimuli on bone regeneration [23, 28, 29], mechanical regulation of tissue differentiation and remodeling [30–32], and fluid–structure interactions of bone marrow within trabecular bone [33, 34]. The non-destructive nature of microCT further makes it ideal for longitudinal evaluation of disuse- and mechanical load-induced bone remodeling and adaptation [35, 36]. For example, Müller and colleagues [37–39] have recently published a series of articles using time-lapse *in vivo* microCT and FE analysis in a mouse tail vertebra model to longitudinally evaluate the effect of compressive loading or unloading on local bone formation and resorption (Figure 4). Locations of bone formation and resorption correlated with sites of high- and low-strain energy density, respectively, and bone modeling/remodeling did not exhibit a ‘lazy zone’ as predicted by the long-accepted Frost mechanostat theory [40].

Space limitations prevent comprehensive discussion of the many applications of microCT to bone biomechanics and mechanobiology. We refer interested readers to several excellent focused reviews [2, 41, 42].

## Tissue engineering

MicroCT emerged as a commercially available tool in the middle of the ‘go-go’ years of tissue engineering (that is, the 1980s and 1990s) [43], positioning it perfectly for widespread use as the problems targeted by tissue engineers necessitate non-destructive, 3D, quantitative imaging techniques. Tissue engineering approaches have remarkable potential to regenerate damaged and diseased tissues, but increasing evidence highlights the need for control of biomaterial properties to meet the biomechanical and biological requirements of complex tissues and organs. Scaffolds must balance mechanical properties with degradation kinetics and byproducts, sufficient porosity for cellular infiltration and seeding, and drug delivery characteristics, among other criteria [44]. Thus, non-destructive quantification of microstructural characteristics such as porosity, surface-to-volume ratio, interconnectivity, and anisotropy is necessary for scaffold optimization [1, 45], and microCT has the potential to provide comprehensive data on these parameters [46].

Scaffold porosity and pore interconnectivity are key factors in biomaterial design to enable cell migration, proliferation, and extracellular matrix production and facilitate tissue in-growth and blood vessel invasion but come with trade-offs in other scaffold parameters, such as mechanical properties [47, 48]. For example, Lin and colleagues [49] used microCT to demonstrate the effect of longitudinal macroporosity and porogen concentration on volume fraction, strut density, and anisotropy in oriented porous scaffolds. MicroCT has become a critical tool for quantitative and non-destructive assessment of internal scaffold microstructure to guide scaffold design and manufacture [50–52] and enables non-destructive evaluation of both microstructural and mechanical behavior of multi-phase and fiber-reinforced scaffolds [51, 53, 54] as well as longitudinal scaffold degradation [55].

MicroCT is also used to evaluate the ability of cell-based tissue engineering bone constructs to form biologic mineralized matrix *in vitro* [56, 57]. These studies and others have demonstrated that osteogenic differentiation of stem cells *in vitro* is dependent on substrate material and microstructural characteristics [58], cell source (for example, amniotic fluid- versus bone marrow-derived mesenchymal cells) [56], and dimensional (that is, 2D versus 3D) [59] and biomechanical culture conditions [60]. Unlike other *in vitro* osteogenesis assays, microCT enables longitudinal quantification of the time course of mineralization in 3D without interfering with cell growth or mineral production [57], an important feature for comparison of various cell sources with different mineralization kinetics [56].

**Figure 5:**
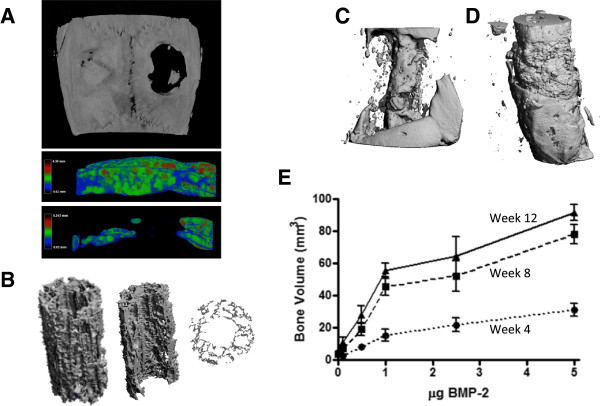
**Microcomputed tomography (microCT) analysis of tissue engineering scaffolds and bone regeneration** ***in vivo*** **. (A)** MicroCT reconstruction of mesenchymal stem cell-mediated bone regeneration in a bilateral cranial defect model treated with a high-density human mesenchymal stem cell (hMSC) construct incorporating growth factor-loaded microparticles (left) or empty control (right), and sagittal-cut views of three-dimensional thickness mapping overlay of defect regions (Phuong Dang and Eben Aslberg, in preparation). **(B)** Poly(L/DL)-lactide tri-calcium phosphate (PLDL-TCP) scaffold created according to the protocol of [38] featuring oriented microporosity and central core showing isometric view and transverse cross-section. **(C)** *In vivo* bone formation in a rat femoral bone defect model implanted with cored scaffolds from (A) loaded with rhBMP-2 in alginate hydrogel (McDermott and collegues, in preparation) or **(D)** hydrogel-mediated delivery of rhBMP-2 within a polycaprolactone nanofiber mesh without a structural scaffold in the defect. Reproduced with permission from [49]. **(E)** *In vivo* microCT-based longitudinal quantification of bone formation over time (dotted to solid lines) for various doses of BMP-2 in the hybrid nanofiber mesh/alginate delivery system. Reproduced with permission from [49].

In addition to scaffold microstructure, microCT enables assessment of tissue engineered bone formation in animal models [61–65] (for example, high-density stem cell-mediated bone regeneration of calvarial defects) (Figure 5A). To evaluate the importance of porosity and space for tissue regeneration, scaffolds were created as described by Lin and colleagues [49], modified by removal of a 1.5-mm diameter axially oriented cylindrical core (Figure 5B), loaded with 3 μg rhBMP-2, and implanted in rat femoral bone defects. Bone formation was localized predominantly to the core space and outer surfaces of the scaffold, indicating a failure of new bone formation to grow into the scaffold itself (Figure 5C), and a hydrogel delivery approach featuring a similar dose of rhBMP-2 (2.5 μg) but without a structural scaffold resulted in greater bone formation, throughout the defect (Figure 5D) [66]. These data suggest that, in spite of high and oriented porosity, structural scaffolds can impede cellular infiltration and tissue regeneration, warranting further research on the role of scaffold porosity and orientation.

Excitingly, multiple companies now provide microCT scanners with a stationary sample container and a rotating gantry housing the x-ray emitter and detector, allowing *in vivo* imaging of small animals (that is, mice and rats) under anesthesia at resolutions approaching those of standard desktop systems. These systems enable longitudinal quantification of scaffold-integration and mineralization. For example, Boerckel and colleagues [66] recently evaluated the time course of bone regeneration in a rat segmental bone defect model over 12 weeks to quantify the dynamics of bone formation, mineralization, and maturation (Figure 5E). Important considerations for *in vivo* microCT imaging include consistent positioning of animals to minimize system variability, volume of interest selection and thresholding to avoid fixation hardware artifacts, and dose of ionizing radiation. As these studies demonstrate, advances in both desktop and *in vivo* microCT imaging systems will continue to further the field of tissue engineering in years to come.

## Vascular imaging

Evaluation of soft tissues by x-ray imaging requires application of radiodense contrast agents. Contrast-enhanced microCT angiography enables visualization of cardiovascular structures, and emerging techniques are enabling this analysis both *ex vivo* and *in vivo*.

### *Ex vivo*microcomputed tomography angiography

**Figure 6:**
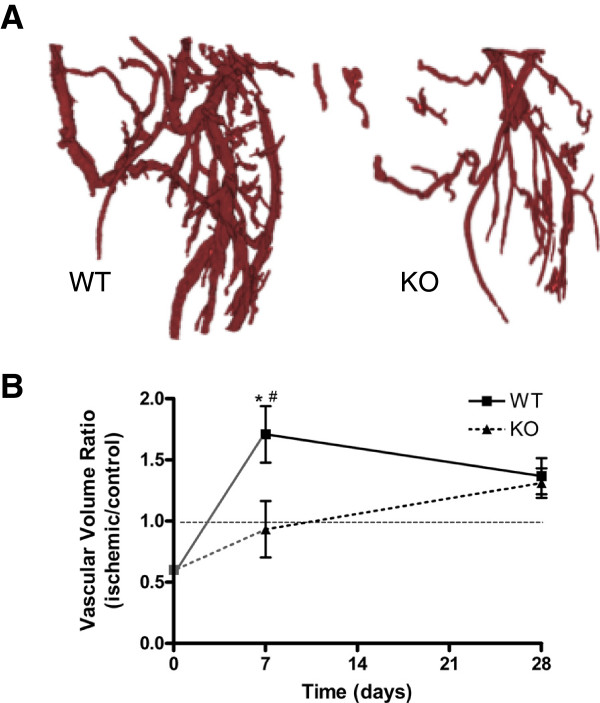
**Microcomputed tomography angiography analysis of hindlimb ischemia recovery. (A)** Three-dimensional reconstructions of distal hindlimb vasculature in wild-type (WT) (left) and MKP-1 knockout (KO) (right) mice at day 7 post-surgery. Reproduced with permission from the American Heart Association [71]. **(B)** Quantification of vascular volume ratio (ischemic/contralateral control), illustrating the biphasic nature of angiogenic and arteriogenic vascular recovery. Initial conditions (T_0_) taken from comparable WT C57Bl6 mice in [75], with continuity indicated by gray lines. Horizontal line illustrates ischemic/control ratio of 1.

**Figure 7:**
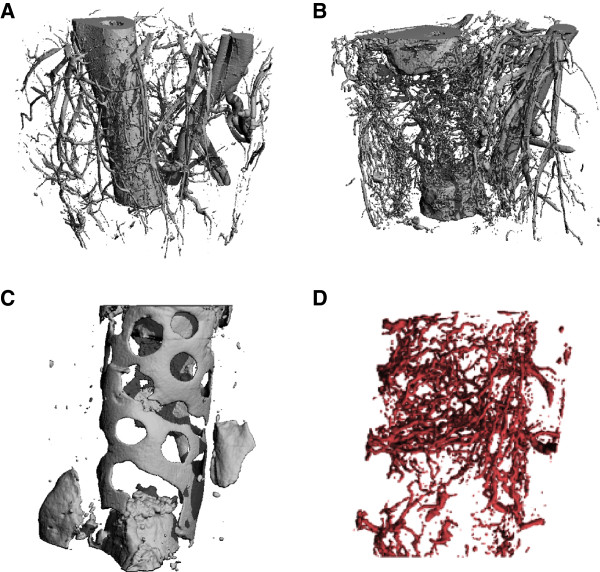
**Microcomputed tomography (microCT) angiography of vascular response to bone injury and regeneration. (A)** MicroCT image of age-matched unoperated rat femur with surrounding vasculature: the large vessels on the right-hand side are the femoral artery and vein. **(B)** Vascular structures and bone ends 3 weeks after creation of an 8-mm bone defect. There is a robust neovascular response to bone injury, characterized by increased branching, new vessel formation, and decreased degree of vascular anisotropy. **(C)** MicroCT image of a nanofiber mesh wrapped around a bone defect. Bone formation has occurred on the surface of the mesh, highlighting the mesh perforations. **(D)** MicroCT angiography was used to visualize radially directed vascular ingrowth from the surrounding soft tissue envelope through the nanofiber mesh perforations, illustrating the contribution of soft tissues to regenerate bone vascularization and the importance of biomaterial porosity. Owing to the overlapping attenuation of bone and the lead chromate contrast agent, separate segmentation within the same sample is not possible without extensive post-processing and image registration. Images reproduced with permission from the National Academy of Sciences [28] (frames A and B) and from Elsevier [66] (frames C and D).

The use of microCT to study 3D vascular morphology began with studies of reno-vascular architecture, in which 3D casting of kidney vessels had been a common visualization approach, dating back to the famed British anatomist Sir William Bowman in the mid-19th century [67]. It was therefore a natural progression from polymeric vascular casting combined with interstitial tissue clearing agents (for example, methyl salicylate) [68] to radiodense contrast-agent casting and CT. High-resolution analysis of model animal vascular structures by microCT was first conducted on renal microvasculature in 1998 [69]. Today, *ex vivo* microCT angiography is a powerful tool for 3D high-resolution evaluation of post-natal vascular growth in models of tissue ischemia (Figure 6) [70, 71], tissue engineering (Figure 7) [28, 72], and tumor angiogenesis [73, 74]. Owing to the small size of arteriole and venule microvasculature and the difficulty of efficient perfusion of potentially leaky vessels in certain animal models, several variables must be considered depending on application, including contrast-agent properties such as radiodensity and viscosity, scanning resolution, threshold and segmentation approaches, and output parameters. Common vascular contrast agents include microfil MV-122 and BaSO4/gelatin. Many of these variables have been systematically addressed in a hindlimb ischemia model [70].

*Ex vivo* microCT angiography has enabled novel observations of fundamental biological processes. For example, serial perfusion and microCT scanning [75] has demonstrated that the process of recovery from hindlimb ischemia is analogous to the response profile of an under-damped feedback control system, with a biphasic recovery featuring an early vessel growth phase resulting in vascular parameters exceeding those of native vessel architecture and a later remodeling phase of vascular rarefaction and remodeling back to normal parameters [71, 75] (Figure 6A). A further advantage of microCT is the ability to separate 3D regions of interest, enabling independent analysis of the upper hindlimb, where arteriogenesis dominates vascular recovery, and the distal hindlimb, where angiogenesis dominates [71]. This approach is limited by resolution, with voxel sizes typically around 20 μm. As capillary beds have vessel diameters of 5 to 10 μm, many vessels will be missed or registered as only partial volumes; therefore, care must be taken during interpretation regarding the detailed processes of angiogenesis and arteriogenesis, and these larger-scale measurements should be confirmed by immunohistochemical analysis on the microvascular scale.

In the context of bone/vascular interactions, the overlap in attenuation coefficients for bone and vascular contrast agents enables simultaneous segmentation of bone and vascular structures (Figure 7A,B) but requires decalcification and volume subtraction for separate quantification (Figure 7D) and precludes bone microstructural analyses (for example, connectivity and density) other than volume [28]. Development of novel contrast agents with non-overlapping attenuation histograms or application of next-generation microCT approaches (for example, spectral CT) would enable simultaneous segmentation of bone and vascularity without decalcification.

### *In vivo*microcomputed tomography angiography

Recent advances in intravenous microCT contrast agents and cardiac and respiratory gating strategies have enabled *in vivo* microCT imaging of cardiac and vascular structures, albeit with reduced contrast, resolution, and quantitative functionality compared to *ex vivo* microCT angiography [76, 77]. Briefly, *in vivo* microCT angiography can be performed by using iodinated monomer-based bolus (for example, iomeprol) or lipid immulsion-based blood-pool (for example, Fenestra VC, MediLumine Inc., Montreal, QC, Canada) contrast agents [76]. Owing to the short cardiac cycle and rapid respiration rate of small rodents, gating strategies (either prospective or retrospective) are required to minimize motion artifacts. In prospective gating, acquisition of images is initiated in response to a physiological signal (for example, electrocardiography); in retrospective gating, physiological signals are recorded at the same time as image data to be sorted later [78]. Prospective gating can have long acquisition time but wide-angular distribution, whereas retrospective gating is characterized by fast scanning and irregular angular distribution [76]. Developing new approaches to achieve high-quality, quantitative *in vivo* microCT imaging remains an active area of research, and the most successful to date have relied on custom microCT or volumetric CT systems capable of rapid scan times and high resolution [76, 77].

## Contrast-enhanced cartilage imaging

**Figure 8:**
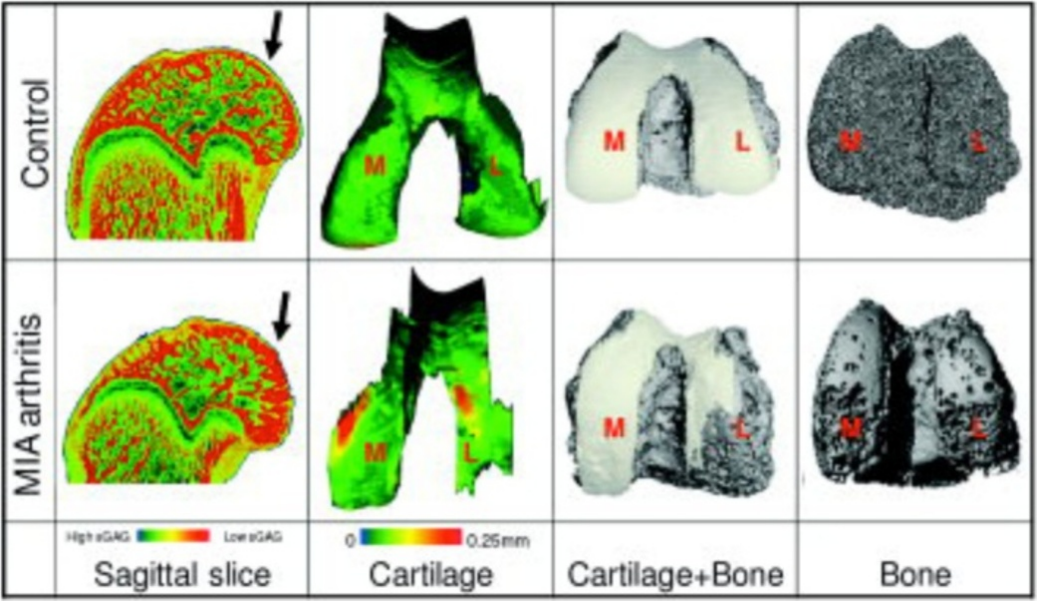
**Equilibrium partitioning by ionic contrast-agent microcomputed tomography (EPIC-microCT).** Experimental osteoarthritis was induced by intra-synovial injection of monosodium iodoacetate (MIA) and evaluated at week 3 by EPIC-microCT. Excised rat femurs were equilibrated with an ionic contrast agent (hexabrix) and scanned to assess cartilage and subchondral bone. The arthritic group exhibited substantial sulfated glycosaminoglycan depletion, cartilage degradation, and subchondral bone resorption, illustrating the capacity of contrast-enhanced microCT to quantitatively assess cartilage and bone in preclinical models of osteoarthritis. Arrows indicate location of complete cartilage degradation in MIA group and corresponding location in the control. L, lateral; M, medial. Figure reproduced with permission from John Wiley & Sons, Inc. [80].

Recently, a radiopaque contrast agent has been developed to enable microCT imaging of non-mineralized cartilage by taking advantage of the charged nature of normal cartilage extracellular matrix. Healthy articular cartilage contains a large amount of negatively charged sulfated glycosaminoglycans (sGAG) such as aggrecan, whereas the interstitial fluid carries positively charged solutes, resulting in net electroneutrality [79]. However, the early stages of osteoarthritis are characterized by a cleavage of these proteoglycans, resulting in reduced sGAG content in the diseased tissue. Palmer and colleagues [79] developed a technique called equilibrium partitioning of ionic contrast agent via microCT (EPIC-microCT), in which the tissue is equilibrated with the radiopaque, negatively charged contrast agent, hexabrix (ioxaglate), which is distributed inversely to the fixed negative charges on the proteoglycan matrix, enabling simultaneous, non-destructive microCT evaluation of both cartilage morphology and composition (Figure 8) [80]. This technique has been applied to evaluate numerous normal and osteoarthritic disease models, including rabbit [79], rat [81, 82], mouse [83], dog [84], and goat [85] as well as human cadaveric cartilage [86]. EPIC-microCT has also been used to non-invasively image cartilage degeneration longitudinally *in vivo* [87, 88], although challenges associated with contrast agent leakage and equilibration time remain. In summary, this technique promises to become a standard in animal model studies of osteoarthritis as it is non-destructive and provides quantitative morphological and compositional outcomes.

## Next-generation approaches

Advanced, composite, and next-generation microCT imaging modalities are an active area of research. One rapidly emerging technique that takes advantage of x-ray intensity spectra instead of integrating attenuation over the entire spectrum is spectral microCT [89]. The theoretic framework for spectral CT was established by Alvarez and Macovski in 1976, when they demonstrated that dual-energy x-ray imaging enables deconvolution of the effects of Compton and photoelectric scattering, the two interactions that contribute to the linear attenuation coefficient [90]. Thus, precise local density mapping can be accurately quantified [90, 91]. This approach further yields 3D information on atomic composition and electron density [92], enables discrimination between materials that would have the same attenuation in standard microCT [89, 93], and dramatically expands the supply of contrast agents [93].

A second, rapidly evolving experimental microCT imaging technique is phase-contrast microCT. This powerful imaging modality bases image detection on the phase shift of refracted x-rays rather than intensity attenuation and promises increased tissue contrast and greater resolution for even soft tissues without the need for contrast agents [94, 95]. For most biological materials, the phase shift of the incident x-ray is proportional to the sample mass density, enabling high-contrast imaging of both soft and hard tissues by microCT [95]. Recently, Tapfer and colleagues, in collaboration with Bruker® MicroCT (Bruker Corporation, Billerica, MA, USA), described a rotating gantry phase-contrast microCT system based on a polychromatic x-ray source [96] that they have applied to *ex vivo* scans of a murine pancreatic tumor model with soft tissue contrast similar to MRI [97]. It is likely that *in vivo* phase-contrast microCT imaging for small-animal models will be established in the coming years, which will represent a great advance in microCT imaging capabilities.

## Conclusions

MicroCT has contributed to dramatic advances in biology and bioengineering over the past 30 years, enabling fundamental studies in bone structure and function, quantitative evaluation of disease progression and treatment, development of new tissue engineering strategies, and contrast-enhanced soft tissue imaging. Both desktop and *in vivo* microCT systems are increasing in availability and application, and continued advancements and innovations promise to continue this trajectory into the future.

## Note

This article is part of a thematic series on *Functional imaging in regenerative medicine*.

## Abbreviations

2D: Two-dimensional
3D: Three-dimensional
BaSO_4_: Barium sulfate
CT: Computed tomography
EPIC-microCT: Equilibrium partitioning of ionic contrast agent via microcomputed tomography
FE: Finite element
HIF: Hypoxia-inducible factor
microCT: Microcomputed tomography
MRI: Magnetic resonance imaging
sGAG: Sulfated glycosaminoglycans.
